# Development of multiepitope subunit protein vaccines against *Toxoplasma gondii* using an immunoinformatics approach

**DOI:** 10.1093/nargab/lqaa048

**Published:** 2020-07-01

**Authors:** Olugbenga S Onile, Glory J Ojo, Bolaji Fatai Oyeyemi, Gbenga O Agbowuro, Adeyinka I Fadahunsi

**Affiliations:** Biotechnology Programme, Department of Biological Sciences, Elizade University, 340211, Ilara-Mokin, Nigeria; Biotechnology Programme, Department of Biological Sciences, Elizade University, 340211, Ilara-Mokin, Nigeria; Molecular Biology Group, Department of Science Technology, The Federal Polytechnic, 360231, Ado-Ekiti, Ekiti State, Nigeria; Biotechnology Programme, Department of Biological Sciences, Elizade University, 340211, Ilara-Mokin, Nigeria; Biotechnology Programme, Department of Biological Sciences, Elizade University, 340211, Ilara-Mokin, Nigeria

## Abstract

Approximately one-third of the world’s human population is estimated to have been exposed to the parasite *Toxoplasma gondii*. Its prevalence is reportedly high in Ethiopia (74.80%) and Zimbabwe (68.58%), and is 40.40% in Nigeria. The adverse effect of this parasite includes a serious congenital disease in the developing fetus of pregnant women. After several efforts to eliminate the disease, only one licensed vaccine ‘Toxovax’ has been used to avoid congenital infections in sheep. The vaccine has been adjudged expensive coupled with adverse effects and short shelf life. The potential of vaccine to likely revert to virulent strain is a major reason why it has not been found suitable for human use, hence the need for a vaccine that will induce T and B memory cells capable of eliciting longtime immunity against the infection. This study presents immunoinformatics approaches to design a *T. gondii*-oriented multiepitope subunit vaccine with focus on micronemal proteins for the vaccine construct. The designed vaccine was subjected to antigenicity, immunogenicity, allergenicity and physicochemical parameter analyses. A 657-amino acid multiepitope vaccine was designed with the antigenicity probability of 0.803. The vaccine construct was classified as stable, non-allergenic, and highly immunogenic, thereby indicating the safety of the vaccine construct for human use.

## INTRODUCTION

Toxoplasmosis is a cosmopolitan zoonotic disease, usually caused by the protozoan parasite *Toxoplasma gondii* that belongs to the phylum Apicomplexa (1). This disease is sometimes asymptomatic, common in immunocompromised individuals, reduces quality of life and usually culminates into severe morbidity (1,2). This parasite affects humans and several warm-blooded animals; it is estimated that about one-third of the world population is infected (3,4). Most prevalent cases of *T. gondii* in Africa are 74.80%, 68.58% and 40.40% from Ethiopia, Zimbabwe and Nigeria, respectively (5).

Incidence at birth of congenitally acquired toxoplasmosis in cases per 10 000 live births was 2.9 and 10–13 for France and Brazil (6,7), respectively. A recent report from meta-analysis showed significantly high occurrence of latent toxoplasmosis in pregnant women, especially in middle- and low-income countries of South America and Africa (8); infected pregnant women are at high risk of miscarriage (9,10). Sulfadiazine and pyrimethamine (Daraprim) combinations are widely administered for the treatment of toxoplasmosis, but this is characterized by various side effects, coupled with high cost and inadequacy (1). In most cases, the drug is only active during the acute stage of infection and may not eradicate encysted parasite (11).

Despite several scientific attempts, there is no existing commercially available vaccine for toxoplasmosis in both humans and animals (12–15). Studies have also reported that inducing Toll-like receptor (TLR) by microbial ligands plays a crucial role in triggering an immune response in *T. gondii* (16,17). Mice injected with killed tachyzoite lysate have also shown no protection against *T. gondii*, neither alone nor when used with an adjuvant (18). More so, an *in silico* approach has suggested that a rhoptry antigen like ROP16 was immunogenic and nonallergenic, thus a target vaccine candidate (13). Nonetheless, there is only one licensed vaccine ‘Toxovax’, which is based on live attenuated tachyzoites of strain S48, that has been approved and used to minimize rate of abortion in sheep, rather than humans (19). A macromolecular-based vaccine has been confirmed to elicit strong humoral and cellular immune responses and is more efficient and safer (20). Thus, research priorities on prevention and treatment of toxoplasmosis could be shifted from chemical drugs and attenuated vaccines to protein vaccines.

In recent years, most scientific research conducted in the identification of vaccine candidates capable of eliciting effective immunity against the disease is focused on molecules of the following protein antigens: surface antigens, dense granule excreted–secreted antigens, rhoptry antigens, microneme antigens, etc. (1,13,21). However, the microneme proteins are particularly promising as vaccine antigens, because they are responsible for host cell recognition, binding, secretion of rhoptry organelles, and cell penetration of all apicomplexans (1,21–22).

The method of host cell invasion by *T. gondii* is complex and consists of several consecutive steps initiated by the release of proteins from secretory organelles called micronemes (MICs) and rhoptries (ROPs) (1,12–13). MICs are specialized secretory organelles in *T. gondii* and some of the proteins including *T. gondii* microneme protein 1 (TgMIC1), TgMIC4 and TgMIC6 usually form a complex that is crucial to host cell invasion (23). Recent studies have supported this claim by showing the importance of TgMIC proteins during the parasite invasion and their ability to induce significantly higher immunogenicity (21,23–25). *Toxoplasma gondii* being a ubiquitous organism can infect a plethora of warm-blooded animals, including mammals and birds. In addition, infection in domestic animals is a threat to public health as toxoplasmosis is a zoonotic disease. There is thus an urgent need to develop vaccines (especially protein vaccine) against *T. gondii*. The use of MIC protein as a vaccine candidate has been reported to be a promising approach due to the protein ability to elicit Th1 immunity and is known to provoke powerful immune responses in mice and humans (21,24–25), hence the choice of MICs in this study as a focus for vaccine development. This research intends to develop multiepitope micronemal protein vaccines against *T. gondii* using an immunoinformatics approach.

## METHODOLOGY

### Retrieval of *T. gondii* micronemal protein (MIC3 gene) sequence for vaccine construction

The amino acid sequences of *T. gondii* micronemal protein were retrieved from the UniProt protein database (www.uniprot.org) and subjected to multiepitope vaccine designing. Retrieved protein sequences were subjected to an antigenicity prediction using the ANTIGENpro database (http://scratch.proteomics.ics.uci.edu), as the main purpose of vaccination is to induce immune response in the host. The potential of ANTIGENpro in predicting potent antigens is well emphasized by Magnan *et al.* (26). The antigenic probability of ≥0.8 was used to determine which proteins to be chosen in the next step of the multiepitope vaccine construction (27).

### The prediction of cytotoxic T lymphocyte and helper T lymphocyte epitopes and immunogenicity

To get an immunogenic cytotoxic T lymphocyte (CTL) epitope capable of inducing cell-mediated immunity and for memory cells, all selected highly antigenic micronemal protein sequences were fed into the freely accessible NetCTL 1.2 server (http://www.cbs.dtu.dk/services/NetCTL/) using the FASTA format at a threshold value for epitope identification score of 0.75 (default score) (28). The CTL epitopes from the inputted protein sequences were predicted by the server based on the training dataset; it also determines the comb scores, epitope length and immunogenicity scores. Only epitopes with a combined score of >0.75 were selected as the CTL epitopes (27,29). The selected CTL epitopes were further subjected to Immune Epitope Design Database (IEDB; www.iedb.org) for MHC class 1 immunogenicity prediction. The immunogenicity score (≥0.2) vaguely indicates the probability of eliciting an immune response (the higher the score, the greater the probability of immune response) (26,28,30).

The prediction of helper T lymphocyte (HTL) epitopes of 15-mer length for mouse allele (H2-IAb, H2-IAd and H2-IEd) was done for the selected micronemal protein of *T. gondii* using the IEDB. The resulting epitopes were ranked based on their IC_50_ values and percentile rank scores assigned to each predicted epitope. Only epitopes with the lowest percentile rank (≤1.5) for MHC class II and having IC_50_ values <50 nM were selected for the design of final multiepitope vaccine (27,30). The lower the percentile rank score, the higher the epitope’s binding affinity for HTL receptors (29).

The 15-mer interferon gamma (IFN-γ) epitopes were predicted using the IFN epitope server (http://crdd.osdd.net/raghava/ifnepitope/scan.php) for the selected top 17 HTL peptides in the FASTA format. The IFN-γ is an example of cytokine known for its ability to stimulate host innate and acquired immune responses, involve in macrophage and natural killer cell activation and also deliver high response to MHC antigens (30). The IFN epitope prediction server uses the motif and support vector machine (SVM) hybrid as an approach for selection and consists of IFN-γ versus other cytokines as a model of prediction. The IFN server used a dataset capable of activating T helper cells through inducing and non-inducing of MHC class II binders (30,31).

### Construction of multiepitope subunit vaccine

To design a vaccine, the ability to induce innate and adaptive immune responses must be considered; the high-scoring CTL and high-affinity HTL epitopes from the recent predictions were used to generate the vaccine candidate sequences. The subunit vaccine must contain both suitable CTL and HTL linkers (32). The different epitopes were joined using GPGPG and AAY linkers that were added at the intraepitope position, thereby linking the HTL and CTL epitopes, respectively (30). Also, a TLR-4 agonist (RS-09; sequence: APPHALS) chosen as an adjuvant was added at the N-terminal end of protein through an EAAAK linker to enhance the immunogenicity of the designed vaccine. An additional 6× histidine (6×His) residue was added to the C-terminal end of vaccine protein as a tag (27,29–30,33–34).

### B-cell epitope prediction for *T. gondii* proteins

B-cell epitopes are known for their vital role in vaccine design; they form specific antigens to which the B lymphocytes bind and are strong determinants in antigen recognition by the host immune system. The ABCpred server (http://www.imtech.res.in/raghava/abcpred/) was used in the prediction of the 14-mer linear B-cell epitopes for the final vaccine construct based on a recurrent neural network at a default threshold of 0.51 (35). The amino acid sequence of the final vaccine construct was inputted into the ABCpred server in the plain format (30). An earlier study by Bergmann-Leitner *et al.* (36) had reported the role of ABCpred in the prediction of viable B-cell epitopes and therefore explained the choice of server in this study.

### Prediction of antigenicity, allergenicity and physiochemical properties of vaccine protein

The ANTIGENpro (http://scratch.proteomics.ics.uci.edu) server was used to predict the antigenicity of the designed vaccine. This uses protein antigenicity microarray data to predict protein antigenicity, with an accuracy of the combined dataset estimated to be 76% based on cross-validation experiments (26).

The allergenicity potential of the designed multiepitope vaccine was determined using the AllerTOP v2.0 (www.ddg-pharmfac.net/AllerTOP) and AllergenFP (http://ddg-pharmfac.net/AllergenFP/) servers. Allergenicity test provides information on the potential of the designed vaccine to induce allergic reactions associated with the immunoglobulin epsilon antibody response (12,13). AllerTOP v2.0 uses amino acid *E*-descriptors, auto- and cross-covariance transformation, and the *k*-nearest neighbors machine learning methods to classify allergens, while AllergenFP identifies allergens and non-allergens using an alignment-free, descriptor-based fingerprint approach (30,37–38).

Several physiochemical parameters of the designed vaccine candidate were all analyzed using the ProtParam server (https://web.expasy.org/protparam/), which include amino acid composition, the theoretical isoelectric point (p*I*) value, instability index, *in vitro* and *in vivo* half-life, aliphatic index, molecular weight and grand average of hydropathicity (GRAVY) parameters (39).

### Prediction of the secondary and tertiary structures of the vaccine construct

The secondary structure of the vaccine protein was predicted using two freely available online servers, namely PSIPRED (http://bioinf.cs.ucl.ac.uk/psipred/) (40) and RaptorX Property (http://raptorx.uchicago.edu/StructurePropertyPred/predict/). Primary amino acid sequences of vaccine protein were directly inputted into the PSIPRED server for protein secondary structure prediction. PSI-BLAST was used to detect sequences that are significantly homologous to the vaccine protein. The RaptorX used a template-free approach through an emerging machine learning model called DeepCNF (deep convolutional neural fields) to predict protein structures from secondary and tertiary structures along with contact map, solvent accessibility, disordered regions and binding sites of the protein sequences (41).

The tertiary structure of the designed multiepitope subunit vaccine was predicted using the I-TASSER (iterative threading assembly refinement) server (https://zhanglab.ccmb.med.umich.edu/I-TASSER/) as described (28). The last five community-wide CASP experiments have ranked I-TASSER as the best server for protein structure prediction (38). The server predicts from amino acid sequences 3D protein structure models and functions using multiple threading alignments, iterative structural assembly simulations and sequence-to-structure-to-function paradigm (41).

### Refinement and validation of the designed vaccine tertiary structure

To improve the predicted 3D model of the designed multiepitope vaccine peptide as previously done elsewhere (30), the output model of I-TASSER server was refined using the GalaxyRefine server (http://galaxy.seoklab.org/) (30,42). This server that is capable of improving both local and global quality of protein structures uses a successfully tested community-wide CASP10-based refinement approach to rebuild and repack the protein side chain and eventually uses a molecular dynamics simulation to relax the final structure. ProSA-web (https://prosa.services.came.sbg.ac.at/prosa.php) was used in the tertiary structure validation to detect potential errors in the predicted tertiary structure. The server provides information on the overall quality score and any problematic parts of the inputted protein structure (43). The refinement output was validated by the PROCHECK principle using the Ramachandran plot obtained from the RAMPAGE server (Mordred.bioc.cam.au.ck/∼rapper/rampage.php). Energetically allowed and disallowed dihedral angles *ψ* and *ϕ* of an amino acid, calculated based on the van der Waals radius of the side chain, can be visualized using the Ramachandran plot (27,30,44).

### Molecular docking of the designed vaccine candidate with TLR-4

The molecular protein–protein docking of the vaccine protein with TLR-4 was done using the ClusPro 2.0 server in order to check for the binding affinity between the vaccine candidate and TLR-4 receptor (29,45). The refined subunit multiepitope vaccine protein was used as the ligand, while TLR-4 PDB file (4G8A) obtained from the RCSB Protein Data Bank was used as a receptor. The output of the ClusPro server was further remodeled using the PyMOL Molecular Graphics System, version 2.0.

### Codon optimization and *in silico* cloning of the designed vaccine candidate

To optimize the designed vaccine expression rate in a proper expression vector, the initial sequences of the protein vaccine were submitted into the Java Codon Adaptation Tool (JCat) server (http://www.prodoric.de/JCat) for codon optimization and reverse translation in the host *Escherichia coli* strain K12. The codon usage of *E. coli* differs from that of the native host *T. gondii* from where the vaccine protein sequences are derived. Three different options were selected in the JCat server: avoid rho-independent transcription, prokaryote’s ribosome binding site and restriction enzyme’s cleavage sites. To assess protein expression level, the output of the JCat that includes codon adaptation index (CAI) and percentage GC content was used. The GC content of sequence that ranges outside 30–70% indicates unfavorable effects on translational and transcriptional efficiencies (46) and an ideal CAI score should be 1.0, while a score of >0.8 is considered a good score (47). Finally, *E. coli* pET-28(+) was used as a vector for cloning of the adapted nucleotide sequence (with NdeI and XhoI restriction sites at the N- and C-terminal, respectively) of the final vaccine candidate provided by the JCat server using the SnapGene tool to ensure vaccine expression (46).

## RESULTS

### Retrieval of *T. gondii* micronemal protein for vaccine construction

To design a multiepitope subunit vaccine, a total of 11 protein sequences, MIC6 (Q9XYH7), MIC1 (O00834), MIC4 (Q9XZH7), MIC8 (Q9BIM7), MIC2 (O00816), MIC15 (Q1PA41), MIC3 (Q9GRG4), MIC16 (B3VQI5), MIC13 (A0A125YWL1) and MIC11 (Q8IT73), of *T. gondii* were retrieved from the UniProt protein database in the FASTA format. Only nine (MIC6, MIC1, MIC4, MIC8, MIC2, MIC15, MIC3, MIC16 and MIC13) had an antigenicity probability score of ≥0.8 after retrieved protein sequences were subjected to ANTIGENpro for antigenicity test and were thus selected for the final vaccine construct (Table 1).

**Table 1.** *T. gondii* micronemal protein sequences as retrieved from the UniProt database with their antigenicity scores

Serial no.	Protein accession no.	Protein name	Antigenicity score	Selected/non-selected
1	Q9XYH7	MIC6_TOXGO	0.940	Selected
2	O00834	MIC1_TOXGO	0.921	Selected
3	Q9XZH7	MIC4_TOXGO	0.966	Selected
4	Q9BIM7	MIC8_TOXGO	0.948	Selected
5	O00816	TOXGO micronemal protein MIC2	0.906	Selected
6	Q1PA41	TOXGO micronemal protein 15	0.905	Selected
7	Q9GRG4	TOXGO MIC3	0.957	Selected
8	B3VQI5	TOXGO micronemal protein 16	0.920	Selected
9	A0A125YWL1	TOXGM microneme protein MIC13	0.966	Selected
10	Q8IT73	TOXGO microneme protein TgMIC11	0.604	Non-selected

### Predicted CTL and HTL epitope prediction and immunogenicity

A total of 169 CTL (9-mer) epitopes were predicted for the selected nine MIC protein sequences with high antigenicity scores using the CTL 1.2 server that was set at a default threshold score for epitope prediction. Only 25 epitopes with high immunogenicity scores were chosen as the final CTL epitopes to undergo vaccine designing (Table 2). The IEDB MHC class II epitope prediction module was used for the prediction of the HTL epitope, where all the selected nine MIC protein sequences were subjected to the module. High-binding MHC class II epitopes for mouse alleles used for the prediction were H2-1Ad, H2-1Ed and H2-1Ab. A total of 17 high-binding HTL epitopes with a lower percentile score of ≤1.5 with MHC class II were selected and used for the final multiepitope vaccine designing (Table 3). Also, all predicted 17 HTL epitopes were found to have the capacity to induce IFN-γ due to the positive score obtained from the output of IFN epitope server (Table 4).

**Table 2.** The predicted CTL epitopes with their immunogenicity scores for *T. gondii*

Serial no.	Accession ID	Epitopes	Combined score	Length	Immunogenicity score
1	O00834	NVEVAWRCY	1.1106	9	0.33283
		YTEEEGIRQ	0.8811	9	0.40521
2	Q9XZH7	ATDVETVFE	0.7812	9	0.28602
		PLCTVFQWY	0.9631	9	0.20362
3	Q9BIM7	SSIIYHDEY	2.4232	9	0.28119
		YTEVFVNGK	1.2001	9	0.24124
4	Q1PA41	LVAAFIALF	0.8145	9	0.31789
		TSGFRARVY	1.1253	9	0.28383
		TIDWTAHAV	0.8999	9	0.35488
		SSGTIPAGY	1.7344	9	0.22204
		EVEIVGAFY	2.0014	9	0.33994
		YVIEHGSQY	1.1636	9	5e−05
		CSTDEHHFV	0.8824	9	0.25857
		STDEHHFVL	1.6934	9	0.29282
		STTEGAAAY	2.4358	9	0.23906
		WMAIPEGAY	1.5304	9	0.28153
		LTDFVENPV	1.1848	9	0.24751
		GQCHIPEEY	0.8668	9	0.27721
		YTEWSEWST	0.8537	9	0.27882
5	B3VQI5	SCHESWFTY	0.7856	9	0.28014
		YTTVTWQEW	0.7507	9	0.26119
		YSDFADWST	1.6614	9	0.27426
6	A0A125YWL	ETDIVGGRI	1.2262	9	0.27206
		QTAEWRCYF	1.1870	9	0.33021
		STHHITWTK	0.8564	9	0.41881

**Table 3.** HTL epitopes with their percentile ranks for *T. gondii*

Serial no.	Allele	Seq. no.	Start	End	Peptide	Method	Percentile rank	IC_50_
1	H2-IAd	5	2845	2859	QRVPRASLAAQRSTC	Consensus(smm/nn)	0.10	500
2	H2-IAd	5	2844	2858	QQRVPRASLAAQRST	Consensus(smm/nn)	0.24	449
3	H2-IAd	5	2843	2857	TQQRVPRASLAAQRS	Consensus(smm/nn)	0.39	310
4	H2-IAb	5	307	321	DVTHAFTGNPASTAH	Consensus(smm/nn)	0.53	111
5	H2-IAb	5	306	320	FDVTHAFTGNPASTA	Consensus(smm/nn)	0.54	110
6	H2-IAb	5	308	322	VTHAFTGNPASTAHR	Consensus(smm/nn)	0.61	127
7	H2-IAb	5	139	153	IPAGYVWSQSFSAWE	Consensus(smm/nn)	0.81	98
8	H2-IAb	5	140	154	PAGYVWSQSFSAWED	Consensus(smm/nn)	0.86	99
9	H2-IAb	5	138	152	TIPAGYVWSQSFSAW	Consensus(smm/nn)	0.90	98
10	H2-IAb	5	2225	2239	LPFIFQVSTASGTSP	Consensus(smm/nn)	0.92	138
11	H2-IAb	5	137	151	GTIPAGYVWSQSFSA	Consensus(smm/nn)	0.93	96
12	H2-IAb	6	719	733	GASYHYYLSSSVGSP	Consensus(smm/nn)	0.93	122
13	H2-IAb	4	666	680	SVSMIPSAPAPPPSG	Consensus(smm/nn)	0.96	108
14	H2-IAb	5	2224	2238	ELPFIFQVSTASGTS	Consensus(smm/nn)	0.96	137
15	H2-IAb	5	2267	2281	MNALAFEASASQTSI	Consensus(smm/nn)	0.97	192
16	H2-IAb	6	720	734	ASYHYYLSSSVGSPS	Consensus(smm/nn)	0.98	122
17	H2-IAb	5	2266	2280	VMNALAFEASASQTS	Consensus(smm/nn)	1	194

**Table 4.** Predicted IFN-γ inducing capability test for protein HTL epitopes

Serial no.	Epitope	Method	Result	Score
Epitope_1	QRVPRASLAAQRSTC	SVM	Positive	0.48389026
Epitope_2	QQRVPRASLAAQRST	SVM	Positive	0.51034675
Epitope_3	TQQRVPRASLAAQRS	MERCI	Positive	4
Epitope_4	DVTHAFTGNPASTAH	SVM	Positive	0.42424489
Epitope_5	FDVTHAFTGNPASTA	SVM	Positive	0.37597767
Epitope_6	VTHAFTGNPASTAHR	SVM	Positive	0.44956736
Epitope_7	IPAGYVWSQSFSAWE	SVM	Positive	0.53377138
Epitope_8	PAGYVWSQSFSAWED	SVM	Positive	0.53976784
Epitope_9	TIPAGYVWSQSFSAW	SVM	Positive	0.5609686
Epitope_10	LPFIFQVSTASGTSP	SVM	Positive	0.52712642
Epitope_11	GTIPAGYVWSQSFSA	SVM	Positive	0.52240804
Epitope_12	GASYHYYLSSSVGSP	SVM	Positive	0.40829154
Epitope_13	SVSMIPSAPAPPPSG	MERCI	Positive	1
Epitope_14	ELPFIFQVSTASGTS	SVM	Positive	0.55186668
Epitope_15	MNALAFEASASQTSI	SVM	Positive	0.36757511
Epitope_16	ASYHYYLSSSVGSPS	SVM	Positive	0.4257479
Epitope_17	VMNALAFEASASQTS	SVM	Positive	0.37896923

### The constructed multiepitope subunit vaccine

**Figure 1.**
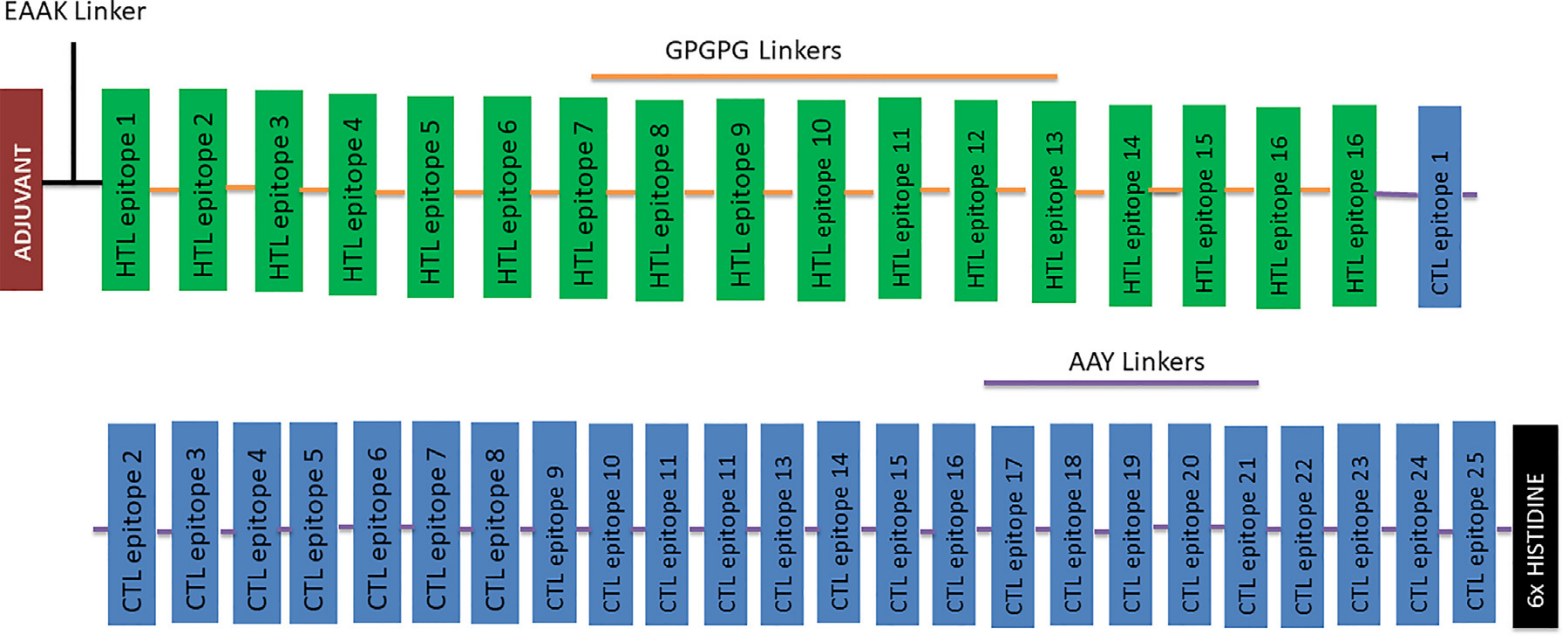
The schematic diagram showing the final multiepitope vaccine protein. The 657-amino acid designed vaccine consists of adjuvant (brown) and HTL (green) epitopes linked by the EAAK linker (black) at the N-terminal end of the protein, where GPGPG (orange) and AAY (purple) linkers were used to join the HTL (green) and CTL (blue) epitopes, respectively. A 6×His (black epitopes) tag is added to the C-terminal end of vaccine protein for identification and purification.

The selected high-scoring CTL and HTL epitopes with high affinity and capacity to induce IFN-γ were used to design the multiepitope subunit vaccine consisting of 17 HTL and 25 CTL epitopes that were linked together with the use of GPGPG and AAY linkers, respectively. An adjuvant was chosen to improve the immunogenicity of the vaccine and was joined by EAAAK linkers to the HTL epitopes at the N-terminal of vaccine protein. At the C-terminal of vaccine, a 6×His tag was added to help in protein purification and identification during *in vivo* experiment. The final vaccine designed consists of 657 amino acid residues merged from 44 epitopes (Figure 1).

### Predicted B-cell epitopes for *T. gondii* proteins

The ABCpred server was used to predict the linear B-cell binding epitopes for the final protein vaccine; a total of 11 epitopes having between 0.87 and 0.92 percentile score with 14-mer length were selected as B-cell epitopes (Table 5). A total of 365 residues located in seven different B-cell discontinuous epitopes with the score ranging from 0.525 to 0.778 were predicted from the final 3D model of the designed protein vaccine.

**Table 5.** B-cell-specific epitopes and their score as predicted by the ABCpred server (the five most ranked epitopes were selected)

Serial no.	Rank	Sequence	Start position	Score
1	1	STAHGPGPGFDVTH	88	0.92
2	1	APPPSGGPGPGELP	266	0.92
3	2	SAWEGPGPGPAGYV	148	0.91
4	3	VTHAFTGNPASTAH	117	0.89
5	4	FYAAYYVIEHGSQY	482	0.88
6	4	ASGTSGPGPGMNAL	287	0.88
7	4	GPGPGSVSMIPSAP	252	0.88
8	4	SFSAWGPGPGLPFI	187	0.88
9	5	TAGPGPGVTHAFTG	110	0.87
10	5	PLCTVFQWYAAYSS	391	0.87
11	5	MNALAFEASASQTS	297	0.87

### The predicted antigenicity, allergenicity and physiochemical parameters of the vaccine construct

The antigenicity of the designed vaccine protein with and without the adjuvant sequences was determined by the ANTIGENpro server and antigenicity probabilities of 0.803091 and 0.825302 were found, which showed the antigenic nature of the designed vaccine with or without the adjuvant, respectively. Antigenicity scores of ≥0.8 for protein sequences were required for a good vaccine construct. All allergenicity tests carried out by both AllerTOP v2.0 and AllergenFP servers for the vaccine construct also showed that the designed vaccine is non-allergenic.

The physiochemical parameters of the vaccine construct showed its molecular weight to be 68.9 kDa and the theoretical p*I* value was 5.42. The half-life was estimated to be 4.4 h in mammalian reticulocytes *in vitro*, >20 h in yeast and >10 h in *E. coli in vivo*. The instability index of vaccine was 35.83, which classifies the protein as stable (instability index >40 indicates instability). The value of the aliphatic index was given as 53.12, while the GRAVY score was −0.184.

### Predicted secondary and tertiary structures of the vaccine construct

**Figure 2.**
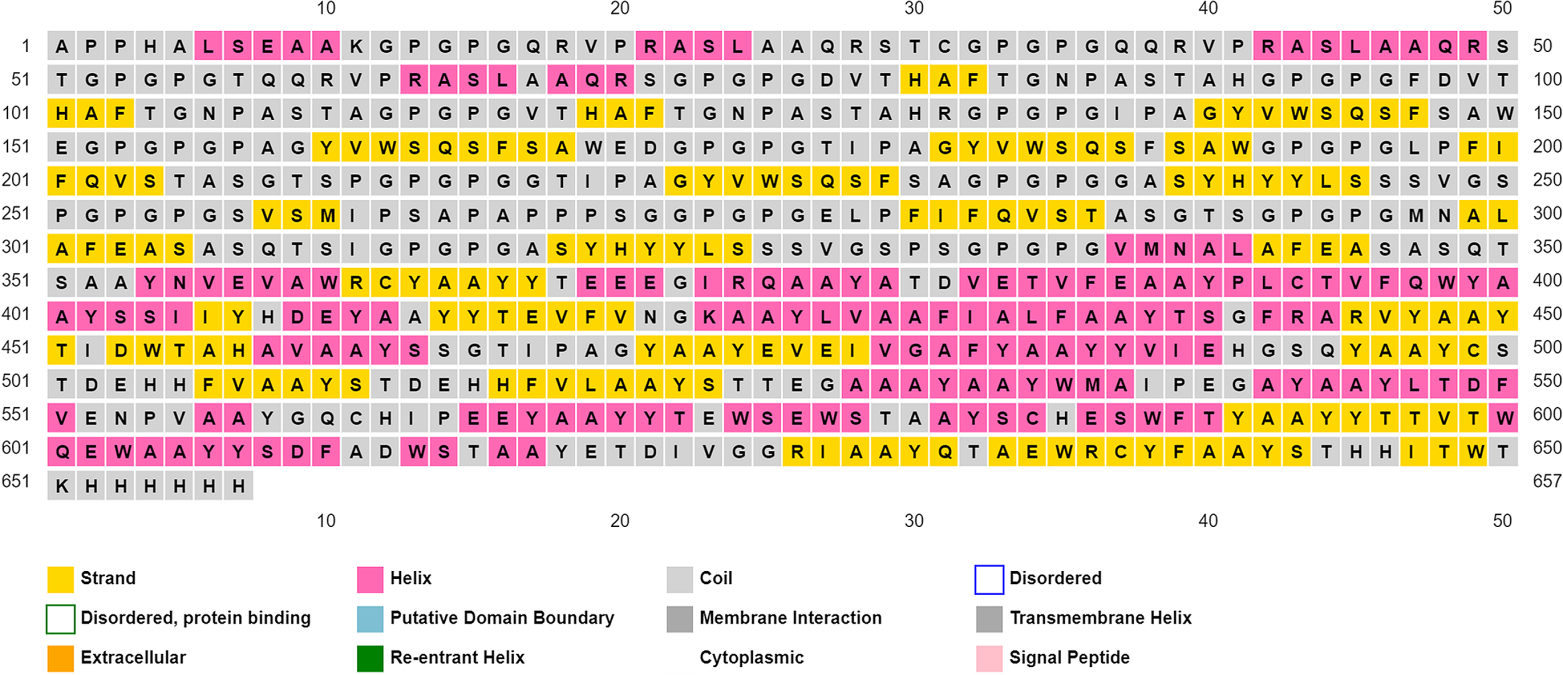
The secondary structure features of the final subunit vaccine sequence. The predicted protein consists of α-helices (14.0%), β-strands (22.0%) and coils (63.0%), while 17% of its positions were predicted as disordered.

**Figure 3.**
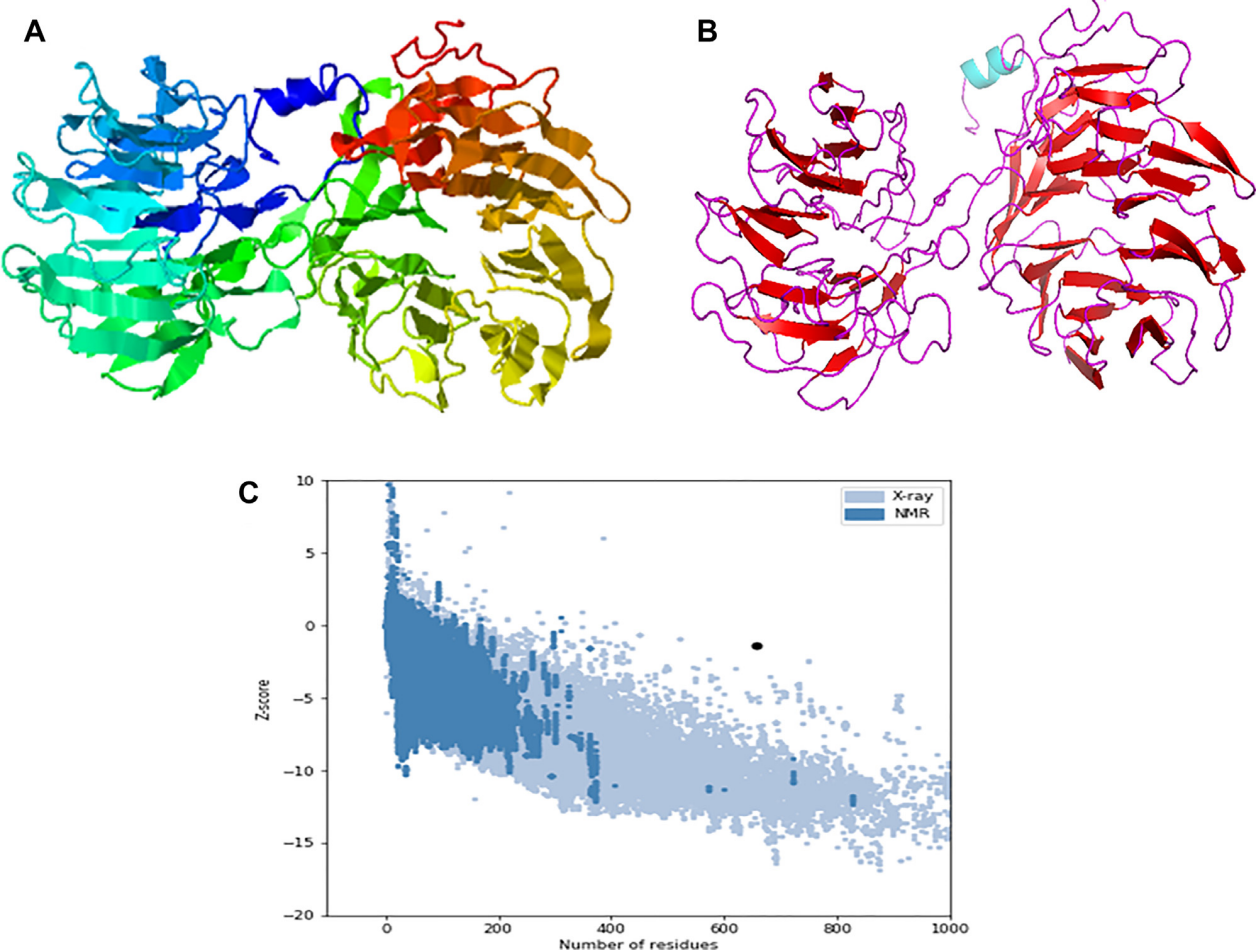
The vaccine protein 3D structure modelling, refinement and validation. (**A**) The crude final 3D model of the multiepitope vaccine obtained from the I-TASSER server before refinement. (**B**) The refined 3D protein vaccine structure as obtained from GalaxyRefine. (**C**) Validation of the refined model with ProSA-web, showing a *Z*-score of −1.38.

The secondary structure of the finally designed vaccine when predicted contained 14% α-helix, 22% β-sheet and 63% coil (Figure 2). Also, the solvent accessibility of vaccine amino acid residues was predicted to be 49% exposed, 18% medium exposed and 32% buried, while a total of 114 (17%) residues were predicted to be in disordered domains as predicted by the PSIPRED and RaptorX Property servers (Figure 2). The tertiary structure of the designed vaccine as predicted by the I-TASSER server showed five best tertiary structure models of the designed vaccine protein using 10 threading templates, which included 1vt4I, 3ixzA, 2pff, 5gs1A and 2pffA. Good alignment with *Z*-score values ranging from 0.31 to 5.54 was found in all the selected 10 templates. The *C*-score values of the best five predicted models ranged from −3.02 to −0.09. The higher *C*-score values indicate higher confidence, with expected range between −5 and 2. The highest *C*-score model was selected for further refinement (Figure 3A). The selected model consists of a Template Model (TM) score of 0.67 ± 0.14 and a root-mean-square deviation (RMSD) score of 10.1 ± 4.6 Å. The scale used for measuring structural similarity between two structures is TM score (48) and it is known to overcome the problem of RMSD, which is sensitive to local error. A TM score (cutoff values are independent of protein length) of >0.5 showed that the model has correct topology, while a TM score of <0.17 indicates a random structural similarity between two structures under consideration.

### Designed vaccine tertiary structure refinement and validation

The refinement of the 3D vaccine structure was done by the GalaxyRefine server that yielded five models. Based on model quality scores for all refined models, among the refined models, model 1 was found to be the best while considering various quality parameters, including GDT-HA (0.9433), RMSD (0.443), MolProbity (2.626), clash score (27.4), poor rotamer score (0.6) and Ramachandran plot score (82.4.5%). The refined model was used as the final vaccine model for further analysis (Figure 3B).

The 3D structure validation was done using the Ramachandran plot analysis of the refined modeled protein and revealed 84.7% residues of vaccine protein were in favored regions, 9.5% of the residues were predicted to be in allowed regions and 5.8% in disallowed regions. ProSA-web was used to determine the quality and potential errors in the crude vaccine 3D model and a *Z*-score of −1.38 was observed (Figure 3C) for the inputted vaccine protein model.

### Molecular docking of MIC subunit vaccine with TLR-4

**Figure 4.**
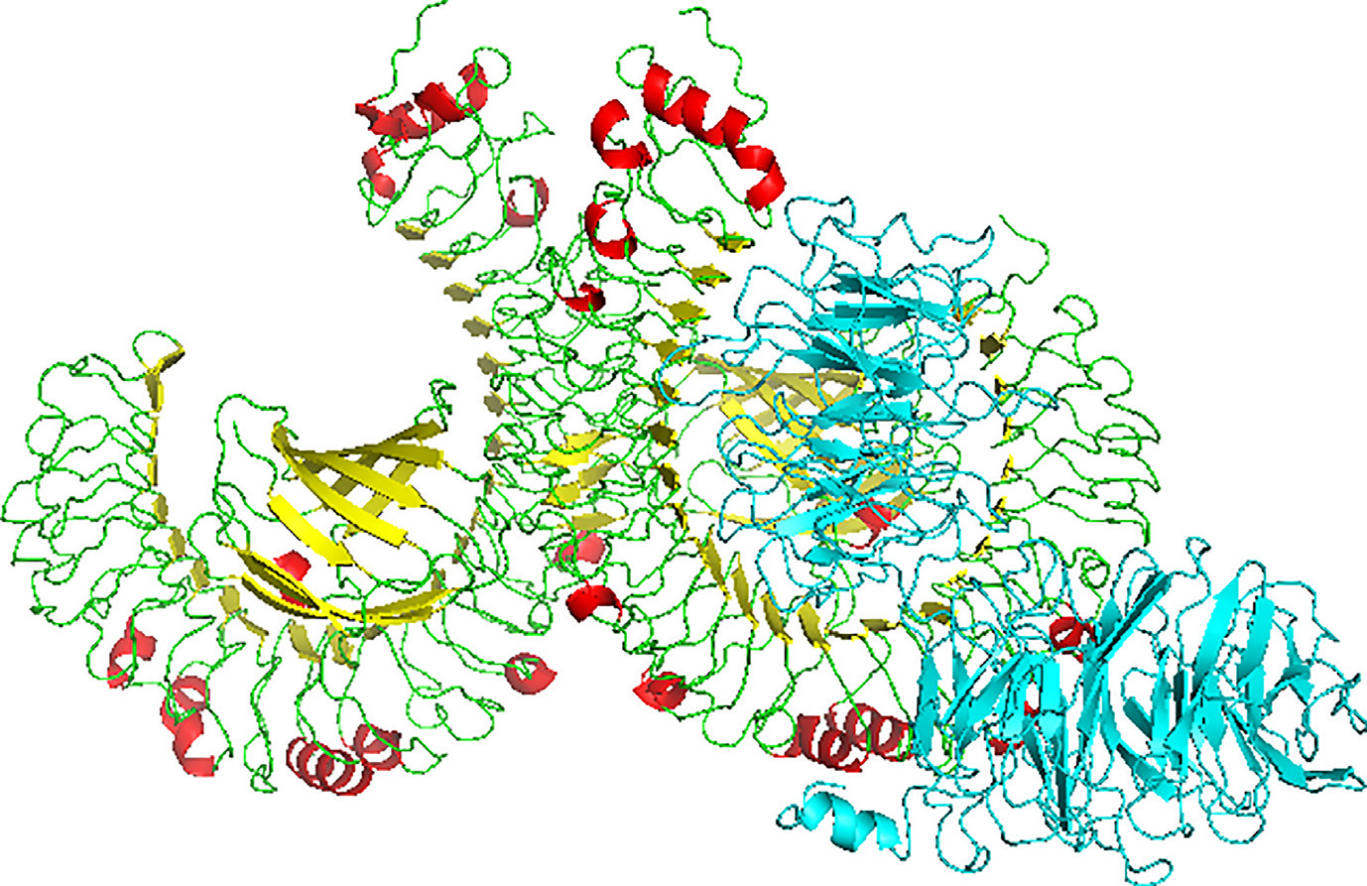
Molecular docking of subunit vaccine with TLR-4 showing the multiepitope subunit vaccine protein (blue) and TLR-4 [helix (red), sheet (yellow) and coil (green)] complex.

The ClusPro 2.0 server was used for the molecular docking between the vaccine construct and the TLR-4, and a total of 30 models were generated from the protein–protein docking complex. Only one model with the lowest energy score of −1375.1 and having the highest binding affinity when compared to other predicted docked complexes fulfilled the desired criteria for the best-docked complex and was eventually chosen (Figure 4).

### Codon optimization of the designed MIC vaccine candidate

JCat was used to optimize codon usage of the vaccine in *E. coli* (strain K12) for maximal protein expression. The length of the optimized codon sequence was 1971 nucleotides, the CAI of the optimized nucleotide sequence was 0.97 and the average GC content of the adapted sequence was 57.1%, thereby showing that the vaccine candidate possibly has good expression in the *E. coli* host. The required percentage range of GC content is between 30% and 70%. Finally, the sequence of the recombinant plasmid was designed by inserting the adapted codon sequences into pET-30a(+) vector using SnapGene software.

## DISCUSSION

About 30% of the world’s population, estimated to be around 2 billion people, is reportedly suffering from at least one neglected tropical disease and most of these diseases are common in low- and middle-income countries (1,8). Considering the lack of effective treatment for parasite eradication by the existing drugs, preventive and/or therapeutic vaccines are needed as long-term solution for at-risk and endemic populations (44,49). Many vaccines against *T. gondii* have been tested with none translated for use in humans and animals (14,15); however, attention has recently shifted toward the development of epitope-based vaccines (12,19,25). Therefore, this research focused on designing a multiepitope subunit vaccine against the parasite *T. gondii* that will contain only the minimal parasite elements necessary to stimulate long-lasting protective or therapeutic immune responses. Epitope-oriented vaccines are characterized with decreased biohazard risk when compared with other types of immunization, ability to engineer and optimize epitope structure to enhance vaccine potential in eliciting strong immunity and ability to focus and generate specific immunity to known conserved immunodominant epitopes (12,19,25,50).

### Retrieval of *T. gondii* micronemal protein sequence for the vaccine construct

The MIC protein selected in this study has been reported to possess the potential of vaccine candidate in immunomic studies (12,21,24–25,51–52). Of the 10 MIC protein sequences retrieved from the database, the nine selected showed high probability for antigenicity test (scores ≥0.8), thereby affirming their good antigenic nature when used for the design of subunit vaccine (12,27,29–30,46).

### Prediction of CTL and HTL epitopes and immunogenicity

The significance of adaptive immunity in human toxoplasmosis is established in patients with acquired or primary defects in T cells; also, mice with deficient B cells, CD4^+^ T cells or CD8^+^ T cells showed increased susceptibility to *T. gondii* infection (17). Epitope-based vaccines that are made of B- or T-cell epitopes have been reported to elicit strong immune responses (12–13,19,25); CTLs (CD8^+^ T cells or killer T cells) kill cancer cells and also kill target cells infected with intracellular viruses, bacteria or protozoa (53). Most cytotoxic T cells have T-cell receptors that can recognize a specific antigen and bind to the MHC class I molecule complex on the antigen, thereby ensuring T-cell destruction of the infected cell (54). In this study, a selection of 25 highly immunogenic CTL epitopes was done for the vaccine design. The selection was based on the ability of epitopes to induce host immune response while considering their immunogenicity scores (12–13,24–25,27).

Roles of helper T lymphocytes in both humoral and cell-mediated immune responses have been well emphasized (27,55). They produce T-cell cytokines that help suppress or regulate immune responses. In this study, 17 highly immunogenic HTL epitopes were selected for the construction of the vaccine protein (30). Nezafat *et al.* (56) have described epitopes specific to HTL receptor as the crucial part of the prophylactic and immunotherapeutic vaccine; these epitopes were found to be capable of inducing the cytokine IFN-γ. Production of IFN-γ has been described as a key component of both innate and adaptive immunity in inhibiting *T. gondii* host multiplication through regulation of antimicrobial responses such as production of nitric oxide and reactive oxygen species, intracellular tryptophan restriction and inducible nitrous oxide synthase expression (57–59). Natural killer, CD4^+^ and CD8^+^ T cells all together will produce large quantities of IFN-γ to expedite the production of protective helper T-cell type 1 immunity against acute and reactivated toxoplasmosis (58).

### Construction of multiepitope subunit vaccine

The multiepitope subunit vaccine protein was predicted from the CTL and HTL epitopes obtained from the selected highly antigenic MIC proteins and joined together with appropriated linkers AAY and GPGPG in order to produce sequences with minimized junctional immunogenicity (29–30,46). Meza *et al.* (60) also reported that spacer sequences played a significant role in the improvement of vaccine design. The bifunctional EAAAK linker used to join the adjuvant and epitope sequences will allow the designed vaccine protein to achieve high level of expression and also ensure that the bioactivity of the fused protein is enhanced (30). Consequently, the designed vaccine with immunogenic CTL and HTL epitopes, appropriate adjuvant and linkers may possess the potential to eliminate and/or inhibit the entry of *T. gondii* parasite in the human host (27,29).

### B-cell epitope prediction for *T. gondii* proteins

B cells function in the humoral immunity component of the adaptive immune system by secreting antibodies (61). They express B-cell receptors on their cell membrane, which allows them to bind to a specific antigen, against which it will initiate an antibody response (61). Having selected high-scoring B-cell epitope (≥0.9) from the designed multiepitope subunit vaccine protein, the vaccine construct may have the potential to induce humoral immunity as well as cell-mediated immune response in humans and animals (12–13,29).

### Prediction of antigenicity, allergenicity and physiochemical parameters of the vaccine construct

The antigenicity probability score of 0.80 obtained in this study represents a true antigenic nature of the vaccine construct and it is comparable with antigenicity probability scores of other previously reported studies (12–13,27,30,46). Pandey *et al.* (27) also emphasized the need for antigenicity test for the vaccine construct as human-based vaccine must be capable of eliciting significant humoral immunological response that eventually results in the formation of memory cells against epitopes of infectious agents. Allergic conditions are caused by hypersensitivity of the immune system to typically harmless substances in the environment; this can cause allergy symptoms like sneezing, inflammation, coughing and allergic asthma, among others. The allergenicity score of the vaccine construct designed here shows that it is a non-allergen to humans (12–13,29,46).

The molecular weight of 68.9 kDa obtained in this study favors the antigenicity of vaccine candidate (13,27), though >18.974 kDa was reported recently by Foroutan *et al.* (12). The predicted theoretical p*I* value reported here (5.42) showed that the vaccine construct is acidic in nature; this is similar to 6.65 reported by Foroutan *et al.* (12) but different from 9.30 reported by Ghaffari *et al.* (13). Also, the stability index of vaccine protein classified it as stable upon expression, thereby enhancing its potential for use and a negatively predicted GRAVY score (−0.184) [though slightly different from −0.290 reported by Ghaffari *et al.* (13)] indicates that the protein is hydrophilic in nature and will interact with water (30,46). The estimated value of aliphatic index presents the designed vaccine as thermostable in nature due to the high value of aliphatic index (13,46). All the predicted physiochemical parameters of the designed vaccine showed that it is immunogenic, thermostable and hydrophilic in nature; these parameters are similar to earlier reports (12–13,30). Thus, the designed vaccine might be useful and suited for use in endemic communities.

### Predicted secondary and tertiary structures of the vaccine construct, its refinement and validation

Meza *et al.* (60) emphasized the importance of the knowledge of secondary and tertiary structures of a protein during protein vaccine designing. Dominance of coils (63%) and 17% disorder were observed in the secondary structure of protein. Structural antigens are known to have natively unfolded regions and α-helical coiled peptides that have the ability to fold into their native structure and become sensitive to naturally induced antibodies during infection (30,62). Vaccine candidate refinement based on the Ramachandran plot prediction showed remarkable improvement in protein structure; a desirable property with 84.7% of residues was found in favored and allowed regions, while fewer residues of vaccine protein were within the disallowed regions, therefore indicating that the overall model of vaccine protein is satisfactory. This is in congruence with a recent report by Ghaffari *et al.* (13).

### Vaccine candidate molecular docking with TLR-4

Studies have reported the role of TLRs, including TLR-4, in protective immunity against *T. gondii* infection (16,63). In this study, only the vaccine–TLR-4 model complex with the highest binding affinity (energy score of −1375.1) was selected during the formation of protein–protein docking complex. TLR-2, -4, -9 and -11 have well been implicated in the immunity against *T. gondii* (17). During oral infection with *T. gondii*, bacterial antigens will translocate from the gut resulting in TLR-2, -4 and -9 reacting to these microbial insults and thus trigger the development of the Th1 immune response (12,64). Also, TLR-2 and -4 are reportedly known to recognize glycosylphosphatidylinositols on the surface of the parasite *T. gondii* (65). TLR played a role in innate immune response production of IL-12 during toxoplasmosis, which allows the human host parasite recognition during infection (17).

## CONCLUSION

Novel control measures will be required if the incessant urge to eliminate *T. gondii* is to be achieved. This will include the discovery and development of candidate vaccine. This study therefore employed the use of several immunoinformatic tools to design multiepitope subunit vaccine with different T-cell (CTL and HTL) and B-cell epitopes against toxoplasmosis, while the focus of vaccine design was placed on very important MIC proteins that are expressed by parasites during host invasion. The understanding of the role of MIC protein in host invasion makes its peptides strategic for both prophylactic and therapeutic benefits and these peptides could eventually be useful in achieving eradication of *T. gondii* infection in endemic areas.
